# Noninvasive Lung Cancer Subtype Classification Using Tumor-Derived Signatures and cfDNA Methylome

**DOI:** 10.1158/2767-9764.CRC-23-0564

**Published:** 2024-07-16

**Authors:** Shuo Li, Wenyuan Li, Bin Liu, Kostyantyn Krysan, Steven M. Dubinett

**Affiliations:** 1 Department of Pathology and Laboratory Medicine, David Geffen School of Medicine, University of California at Los Angeles, Los Angeles, California.; 2 Department of Medicine, David Geffen School of Medicine, University of California at Los Angeles, Los Angeles, California.; 3 Jonsson Comprehensive Cancer Center, University of California at Los Angeles, Los Angeles, California.; 4 VA Greater Los Angeles Health Care System, Los Angeles, California.; 5 Department of Molecular and Medical Pharmacology, David Geffen School of Medicine, University of California at Los Angeles, Los Angeles, California.

## Abstract

Accurate diagnosis of lung cancer is important for treatment decision-making. Tumor biopsy and histologic examination are the standard for determining histologic lung cancer subtypes. Liquid biopsy, particularly cell-free DNA (cfDNA), has recently shown promising results in cancer detection and classification. In this study, we investigate the potential of cfDNA methylome for the noninvasive classification of lung cancer histologic subtypes. We focused on the two most prevalent lung cancer subtypes, lung adenocarcinoma and lung squamous cell carcinoma. Using a fragment-based marker discovery approach, we identified robust subtype-specific methylation markers from tumor samples. These markers were successfully validated in independent cohorts and associated with subtype-specific transcriptional activity. Leveraging these markers, we constructed a subtype classification model using cfDNA methylation profiles, achieving an AUC of 0.808 in cross-validation and an AUC of 0.747 in the independent validation. Tumor copy-number alterations inferred from cfDNA methylome analysis revealed potential for treatment selection. In summary, our study demonstrates the potential of cfDNA methylome analysis for noninvasive lung cancer subtyping, offering insights for cancer monitoring and early detection.

**Significance:**

This study explores the use of cfDNA methylomes for the classification of lung cancer subtypes, vital for effective treatment. By identifying specific methylation markers in tumor tissues, we developed a robust classification model achieving high accuracy for noninvasive subtype detection. This cfDNA methylome approach offers promising avenues for early detection and monitoring.

## Introduction

Lung cancer is the leading cause of cancer-related deaths globally (1) and is characterized by a wide range of clinicopathologic features (2). The different histologic subtypes of lung cancer exhibit variances at the molecular, pathologic, and clinical levels (3), underlining the significance of precise subtype classification for effective diagnosis, monitoring, and prognosis. Accurate classification profoundly impacts the selection of optimal treatment options and patient outcomes during different courses of lung cancer management (4, 5). In the initial diagnosis, the histologic subtype provides critical information about tumor characteristics, guiding treatment selection. During the course of treatment, the development of different histologic tumor profiles can indicate tumor resistance to administered therapies or a new second primary tumor (6, 7). Histologic subtyping of lung cancer is thus essential to tailor initial treatment options as well as longitudinally coordinate cancer management strategies to optimize patient outcomes.

Standard histologic subtyping relies on morphologic, immunophenotypic, and molecular characteristics of tissue specimens collected from tumor biopsies (8). Liquid biopsy, specifically plasma cell-free DNA (cfDNA), has recently demonstrated the potential as a minimally invasive method for cancer diagnosis, typing (9, 10), subtyping (11), and monitoring (12, 13). Unlike a tumor biopsy from a single tumor region, cfDNA is released into the bloodstream by tumor cells from various regions, potentially offering a more comprehensive tumor profile (14, 15). cfDNA therefore represents an alternative method that allows for repetitive monitoring to assess the molecular characteristics of tumors.

In this study, we investigated the potential of differentiating lung cancer histologic subtypes utilizing cfDNA methylome profiles generated by a cost-effective assay, *cfMethyl-Seq* (9). We classified the two most prevalent subtypes of lung cancer, lung adenocarcinoma and lung squamous cell carcinoma (LUSC), which represent 42% to 51% and 17% to 26% of total lung cancer cases, respectively (8). Subtype-specific methylation markers were first systematically identified from high-resolution reduced representative bisulfite sequencing (RRBS) data from 31 lung adenocarcinoma and 29 LUSC tumor tissue samples. The markers were derived at the DNA fragment level overcoming tumor impurity and heterogeneity and capturing methylation patterns present even in minor cell populations (9). We validated the markers’ reproducibility, biological impact, and discriminative power in independent patient cohorts. Based on these tissue-derived markers, we constructed a lung cancer histology classification model from the plasma cfDNA. This is among the *first* cfDNA methylation–based models to noninvasively predict lung cancer subtypes. We evaluated the model performance using cross-validation on *cfMethyl-Seq* data from 106 patients with lung cancer and independent validation on data from 27 patients with lung cancer. Our results demonstrate the potential of noninvasively subtyping lung cancers using cfDNA methylome.

## Materials and Methods

### Data collection

**Figure 1:**
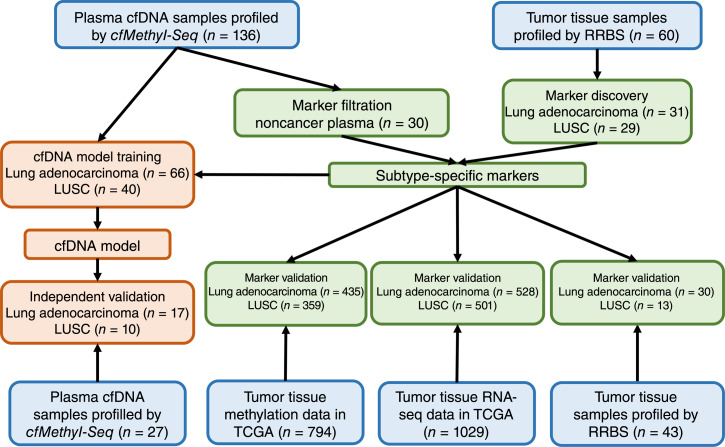
Overview of the data usage and study design. A tissue RRBS dataset (*n* = 60) was used to identify subtype-specific methylation markers. We filtered the markers that were not differential between the solid tumor tissues and the noncancer plasma samples (*n* = 30). The remaining subtype-specific markers were then validated using an independent tissue RRBS dataset (*n* = 43), TCGA project’s tumor tissue methylation data (*n* = 794), and RNA-seq data (*n* = 1,029). We applied the subtype-specific markers identified from tumor tissue to train and validate the subtype classification model on plasma cfDNA using the *cfMethyl-Seq* data of the patients with lung cancer (*n* = 106). Then we validated this cfDNA-based classification model on an independent *cfMethyl-Seq* dataset (*n* = 27).

We generated two *cfMethyl-Seq* datasets from plasma samples, one for marker discovery and model training and the other for independent validation. The first dataset consists of 136 plasma cfDNA samples from 106 patients with lung cancer (66 lung adenocarcinoma and 40 LUSC samples) and 30 noncancer individuals (9). The second dataset consists of 27 plasma cfDNA samples from 17 patients with lung adenocarcinoma and 10 patients with LUSC. We also generated two RRBS datasets from solid tumor tissue samples for marker discovery and validation. The marker discovery set contains 60 solid tumor samples from an independent cohort of 60 patients with lung cancer (ref. 9; 31 lung adenocarcinoma and 29 LUSC), whereas the validation set contains 43 solid tumor samples from 30 patients with lung adenocarcinoma and 13 patients with LUSC. This study was approved by the Institutional Review Board (IRB) of the University of California at Los Angeles (IRB#19-000618, IRB#19-000230, IRB#19-001488, IRB#16-000659, and IRB#17-000985) and was conducted in accordance with the Belmont Report. All participants provided written informed consent. The clinicopathologic characteristics of these patients with lung cancer are summarized in Table 1 and Supplementary Table S1. These datasets are accessible under accession codes EGAS00001006020 and EGAS00001007717 in the European Genome-Phenome Archive. We collected the DNA methylation data and the RNA sequencing (RNA-seq) data of patients with lung cancer in The Cancer Genome Atlas (TCGA) project. TCGA data served as the external validation data for the differential methylation markers and the classification model of lung cancer subtypes. The usage of these datasets and the study design are summarized in Fig. 1.

**Table 1:** Demographic and clinical characteristics of patients with lung cancer. We collected two plasma cfDNA cohorts and two tumor tissue cohorts from patients with lung cancer. One sample was collected per patient. The plasma cfDNA samples were profiled using *cfMethyl-Seq*; the solid tumor samples were profiled using RRBS. The donors in plasma cfDNA cohorts are independent of those in the tissue cohort for marker discovery

	Plasma cfDNA		Solid tumor	
	Model training	Independent validation	Marker discovery	Marker validation
*N*	106	27	60	43
Age (range)	63.5 (32–81)	67 (51–84)	70.5 (44–82)	71 (44–86)
Gender (%)				
Female	28 (26.4%)	7 (25.9%)	28 (46.7%)	20 (46.5%)
Male	56 (52.8%)	19 (70.4%)	32 (53.3%)	23 (53.5%)
Unknown	22 (20.8%)	1 (3.7%)	0 (0.0%)	0 (0.0%)
Stage (%)				
I	27 (25.5%)	10 (37.0%)	35 (58.3%)	24 (55.8%)
II	19 (17.9%)	4 (14.8%)	9 (15.0%)	12 (27.9%)
III	24 (22.6%)	7 (25.9%)	11 (18.3%)	6 (14.0%)
IV	36 (34.0%)	5 (18.5%)	2 (3.3%)	0 (0.0%)
Unknown	0 (0.0%)	1 (3.7%)	3 (5.0%)	1 (2.3%)
Histology (%)				
Lung adenocarcinoma	66 (62.3%)	17 (63.0%)	31 (51.7%)	30 (69.8%)
LUSC	40 (37.7%)	10 (37.0%)	29 (48.3%)	13 (30.2%)

### *cfMethyl-Seq* and RRBS data preprocessing

Three steps were performed to preprocess *cfMethyl-Seq* data. In Step 1, we removed the unique molecular identifier (UMI) sequence and trimmed the raw sequencing reads. Our custom adapters contain an 8-bp random UMI and a 5-bp fixed sequence at the beginnings of both forward and reverse reads. These sequences are removed before adapter trimming (and written into the read name). Trim Galore! (16) was then used to trim the default Illumina adapters from the sequencing reads (using the options –three_prime_clip_R1 15 –three_prime_clip_R2 13 –clip_R2 2 –length 15 –phred33). In Step 2, we performed sequence alignment, deduplication, and methylation calling. Bismark (17) was first used to align the trimmed reads to the reference genome hg19 [ref. 18; GRCh37 (GCA 000001405.1)]. Umi-Grinder (19) removed PCR duplicates based on the UMI labels (now in the read names), allowing four mismatches in the total 16-bp UMI. Bismark methylation extractor was then used to call methylation in the mapped, deduplicated reads. In Step 3, the paired reads R1 and R2 were merged to form one fragment based on their mapping location. Tissue RRBS samples were preprocessed in the same manner as *cfMethyl-Seq* data. *cfMethyl-Seq* is a generalization of RRBS for the cfDNA (9). *cfMethyl-Seq* and RRBS utilize the same restriction enzyme (MspI) and thus enrich the same CpG-dense regions (9, 20). Based on the experimental protocols, we defined the unit regions in our methylation analyses to be the genomic regions (*n* = 1,089,395) that are between two adjacent MspI cutting sites (i.e., between two CCGG sites) and that are less than 350 bp (the average region size is 117 bp). These regions were the enriched regions of the *cfMethyl-Seq* and RRBS. The sequenced DNA fragments from these two experiments should be exactly mapped to these regions.

### Preprocessing of TCGA methylation data

Methylation data in the TCGA project (435 lung adenocarcinoma and 359 LUSC samples) were generated using Illumina Infinium HumanMethylation 450K microarray. These data contained the methylation level (i.e., β value) at predefined CpG sites (i.e., probes), which were different from the enriched regions in the *cfMethyl-Seq* and RRBS. To utilize these data to validate the differentially methylated markers, we mapped the probes of the 450K microarray to the enriched regions of the *cfMethyl-Seq* and RRBS. If a probe targeted a CpG site which fell into an enriched region, the probe was assigned to that region. As a result, the 450K probes could be mapped to approximately 15.1% enriched regions (*n* = 164,314). Considering the pervasiveness of DNA methylation, we approximated the methylation level of an enriched region as the average methylation level of all probes assigned to that region.

### Fragment-based marker discovery between lung adenocarcinoma and LUSC

Tumor-derived methylation alterations may only be present in a small fraction of the DNA molecules in a solid tumor sample because of contaminating noncancer cells (ref. 21; e.g., tumor-associated normal epithelial and stromal cells, immune cells, and vascular cells) and tumor heterogeneity (ref. 22; i.e., presence of the alteration within only a subpopulation of the tumor cells). Conventional methods for methylation marker discovery rely on the averaged methylation level, i.e., β values, defined as the fraction of methylated alleles of all alleles mapped to a genomic region in a sample (23–25). These methods intrinsically blur the differential signals by including the background alleles that are invariant between subtypes (ref. 9; e.g., lung adenocarcinoma and LUSC). Thus, they are insensitive to the differential methylation signals present only in a small proportion of DNA fragments. To overcome impurity and heterogeneity in the tumor samples, we used a fragment-based marker discovery framework to stratify subtype-specific DNA fragments from background DNA fragments (subtype-invariant DNA fragments) to capture tumor subtype signals in a sensitive and specific manner (9). We utilized our previously proposed concept of α value, defined as the percent of methylated CpGs of all CpGs on a DNA fragment (9). This concept has been adopted in several studies to identify cancer-specific methylation markers (9, 25, 26). In brief, we compared samples from two subtypes at the fragment level, by the α values of individual fragments mapped to a genomic region (i.e., the α-value distribution of each sample, Supplementary Fig. S1A). Taking lung adenocarcinoma–specific hypomethylation marker discovery as an example, we identified regions where the α-value distributions of the lung adenocarcinoma samples have a well-separated hypomethylated component from those of the LUSC samples. Our marker discovery method then automatically determines an α-value threshold, i.e., $\alpha_{\text{hypo}}$, in which fragments with α values $\leq \alpha_{\text{hypo}}$ are defined as hypomethylated fragments. Given an $\alpha_{\text{hypo}}$, if the number of lung adenocarcinoma samples with hypomethylated fragments ($n_{\text{hypo}}^{\text{LUAD}}$) is significantly larger than the number of LUSC samples with hypomethylated fragments ($n_{\text{hypo}}^{\text{LUSC}}$), then this genomic region carries significant tumor signals (Supplementary Fig. S1B). If there exists an $\alpha_{\text{hypo}}$ for a genomic region that satisfies $n_{\text{hypo}}^{\text{LUSC}}<2$, we considered this genomic region as a candidate lung adenocarcinoma–specific hypomethylation marker. In this case, the more lung adenocarcinoma samples with hypomethylated fragments, the more robust a candidate lung adenocarcinoma–specific hypomethylation marker. Therefore, we ranked all candidate lung adenocarcinoma–specific hypomethylation markers by $n_{\text{hypo}}^{\text{LUAD}}$. As these methylation markers would be used to distinguish patients with lung adenocarcinoma and LUSC based on their cfDNA, we further required the candidate markers to be differential between 60 lung tumor samples (i.e., lung adenocarcinoma and LUSC) and 30 plasma samples from noncancer individuals. Candidate markers were removed if more than 20% of noncancer plasma samples contained hypomethylated fragments. This filter ensured that the subtype-specific methylation signals were unlikely to be observed in the noncancer plasma samples. The subtype-specific hypomethylated fragments were thus more likely derived from the tumor rather than the background blood cells. This same principle applies to the lung adenocarcinoma–specific hypermethylation marker discovery, in which an α-value threshold, i.e., $\alpha_{\text{hyper}}$, will be determined and those reads with α values $\geq \alpha_{\text{hyper}}$ are defined as hypermethylated reads. Similarly, we also required $n_{\text{hyper}}^{\text{LUSC}}<2$ for all lung adenocarcinoma–specific hypermethylation markers and ranked them by $n_{\text{hyper}}^{\text{LUAD}}$. Following this procedure, we identified markers using the solid tumor RRBS data. In addition, we switched the roles of the lung adenocarcinoma and LUSC samples in the marker discovery and identified LUSC-specific hypomethylation and hypermethylation markers. We utilized the top 2,500 candidates (ties are included) from all marker discoveries as markers for the remaining analyses, i.e., in total, 10,941 subtype-specific markers.

### Validation of the methylation markers between lung adenocarcinoma and LUSC

Methylation markers were validated by three parameters: reproducibility in independent cohorts, association with subtype-specific transcription activity, and distinguishing power between lung adenocarcinoma and LUSC. To assess reproducibility, we used RRBS data from an independent tissue cohort and 450K methylation microarray data from the TCGA project. Due to the small sample size of the RRBS data, we assessed differential methylation by calculating the fold change of the subtype-specific DNA fragments. The 450K data were generated using an experimental platform distinct from the RRBS data. It covered approximately 32.0% of subtype-specific markers (*n* = 3,506) derived from the RRBS data. Differential methylation in the 450K data was evaluated using Student *t* tests following a Benjamini–Hochberg correction for multiple testing.

To evaluate the downstream effects of subtype-specific markers on subtype-specific transcription activity, we analyzed RNA-seq data of tumor samples from patients with lung adenocarcinoma (*n* = 528) and LUSC (*n* = 501) in the TCGA project. RNA-seq data were processed as the transcript per million (TPM) for each gene. To map the transcription data to the markers, we overlapped the promoter regions [defined by GeneHancer (27)] with the marker regions. If the promoter of a gene was identified to have more than 50% overlap with a marker, we defined the gene to be associated with the marker. A heatmap for the TPM of these marker-associated genes was generated. We also performed differential expression analysis on the TPM of the marker-associated genes between the lung adenocarcinoma and LUSC samples using the limma package (28). A Benjamini–Hochberg adjusted *P* value was calculated for each gene. We defined a gene as differentially expressed if its Benjamini–Hochberg adjusted *P* value was < 0.05. We compared the fraction of differentially expressed genes in the marker-associated genes and the fraction of differentially expressed genes in 500 randomly selected gene sets. An empirical *P* value was calculated based on the rank of the fraction of differentially expressed genes in the marker-associated genes among the random genes.

To evaluate the distinguishing power of the markers, we constructed a logistic regression classifier (with the L2 penalty) to distinguish lung adenocarcinoma and LUSC tumor samples in the TCGA project. The model was trained using the methylation level at the subtype-specific markers which were covered in the 450K data. We set the C parameter to C=0.1, and all other hyperparameters use the default values provided using the Python scikit-learn machine learning package (29). To evaluate the classification results, we performed 10-fold cross-validation on the samples and calculated the AUC.

### Classification of lung adenocarcinoma and LUSC using the methylation profile of the cfDNA

Using the fragment-based marker discovery method, each methylation marker is associated with a threshold, i.e., $\alpha_{\text{hypo}}$ or $\alpha_{\text{hyper}}$, which defines hypomethylated or hypermethylated reads, respectively. As the threshold associated with the methylation markers reflects subtype-specific methylation patterns, we regard those hypomethylated or hypermethylated reads as subtype-specific reads. Specifically, for hypermethylation markers, we can identify those fragments that have α-values $\geq \alpha_{\text{hyper}}$ as hypermethylated reads and normalize the number of these reads by the sample sequencing depth, i.e., $\hat{\text{count}}\left(\text{marker}\right)=10^{9}\frac{\text{count}\left(\text{marker}\right)}{\text{raw}\;\text{read}\;\text{count}\;\text{of}\;\text{the}\;\text{genome}}$. Similarly, for hypomethylation markers, we can identify those fragments that have α-values $\leq \alpha_{\text{hypo}}$ as hypomethylated reads and normalize the number of these reads by the sample sequencing depth.

The subtype-specific signal in cfDNA can be weak and unstable because of the limited sequencing depth of our *cfMethyl-Seq* data (median 22.6×) and the generally low tumor content. To obtain robust features for the subtype classification, we combined multiple individual markers into a merged marker. We performed constrained K-means clustering (30) on four types of markers, namely, lung adenocarcinoma–specific hypermethylation markers, lung adenocarcinoma–specific hypomethylation markers, LUSC-specific hypermethylation markers, and LUSC hypomethylation markers. For each type of marker, we allowed 45 to 55 individual markers to be clustered as a group. We then combined the individual markers within a cluster as a merged marker. By combining individual markers, the merged markers have high read coverage and provide stable subtype-specific signals. For every merged marker, we derived a numerical feature by calculating the average normalized read count at this merged marker and transforming the normalized read count by a logarithm, i.e., $\ln\left(\text{average}\left(\hat{\text{count}}\left(\text{marker}\right)\right)+1\right)$. The logarithmic averaged read counts at all merged markers are concatenated into a feature vector, which is used as the fragment-based methylation profile of the cfDNA sample. We created 219 merged markers (i.e., features) from the 10,941 individual subtype-specific markers that were identified from the solid tumor tissues.

We constructed a logistic regression classifier with the L2 penalty to distinguish plasma samples from patients with lung adenocarcinoma and LUSC using the fragment-based methylation profiles. Note that the fragment-based methylation profile of a cfDNA sample was constructed based on the tissue-derived markers. We set the C parameter to C=0.1, and all other hyperparameters use the default values provided using the Python scikit-learn machine learning package (29). To maximally utilize the available samples in the training, we performed leave-one-out cross-validation (LOOCV) on the samples and calculated the AUC.

### Tumor fraction estimation and tumor copy-number alteration detection in the plasma cfDNA using ichorCNA

We utilized ichorCNA (31) to quantify tumor fraction and detect tumor copy-number alterations from plasma cfDNA. ichorCNA compares the sequencing read coverage in the cfDNA with a reference panel in large genomic bins to estimate the tumor fraction. The hg19 genome was segmented into 1 million bp bins, and a reference panel was created from 30 noncancer plasma samples. We applied ichorCNA to the plasma samples from patients with lung cancer in its default settings. If a plasma sample has a nonzero tumor fraction, we consider it to have a detectable tumor fraction when using ichorCNA. Given that ichorCNA has a detection limit of approximately 3% (31), all samples with undetectable tumor fractions are considered to have a tumor fraction of less than 3%. Using the tumor fraction estimated from ichorCNA, we evaluated the accuracy of lung cancer subtype classification in the plasma samples with detectable and undetectable tumor fractions separately. Tumor copy-number alteration was determined for each bin. After purity adjustment, we consider a log_2_-transformed copy-number ratio >0.3 as a gain and < −0.3 as a loss (32). Chromosome 9p21.3 loss is defined by loss of *CDKN2A/B* and/or *MTAP* genes (33).

### Data availability

The following data are used in this study: the RRBS data of solid tumor tissue samples and the *cfMethyl-Seq* data of plasma samples are accessible in the European Genome-Phenome Archive under accession codes EGAS00001006020 and EGAS00001007717.

## Results

### Identification of methylation markers between lung adenocarcinoma and LUSC

**Figure 2:**
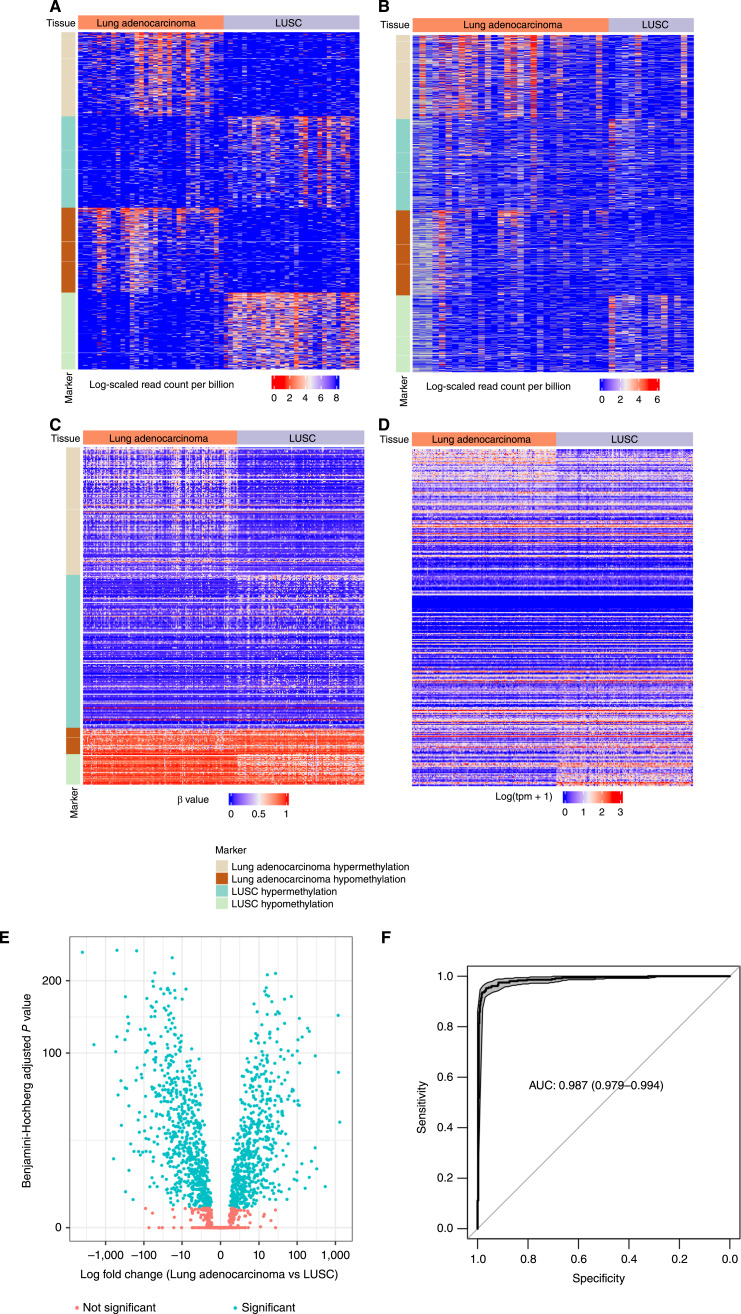
Identification and validation of the subtype-specific methylation markers. **A,** Heatmap of the logarithm-scaled normalized read count at the markers in the tumor tissue RRBS data. For lung adenocarcinoma and LUSC, we selected the top 2,500 hypermethylation and hypomethylation regions using the fragment-based marker discovery method. The color in the heatmap represents the logarithm-scaled normalized count of the hypermethylated (hypomethylated) reads at the hypermethylation (hypomethylation) markers. **B,** Heatmap of the logarithm-scaled normalized read count at the markers in the independent tumor tissue RRBS data. **C,** Heatmap of the methylation levels at the markers in TCGA methylation data. Methylation levels were quantified as the average β value across all probes covered by a marker. **D,** Heatmap of the logarithm-scaled transcription level at the marker-associated genes in the TCGA RNA-seq data. A gene is marker-associated if its promoter has more than 50% overlap with a marker. Every row in the heatmap represents a marker-associated gene, and every column represents a patient with cancer. A transcription level was quantified as the TPM. **E,** Differential gene expression between lung adenocarcinoma and LUSC in TCGA RNA-seq data. The statistical significance of the genes was determined by the Benjamini–Hochberg adjusted *P* values at 0.05. The adjusted *P* values are shown in a negative logarithm (base 10) scale. **F,** The discriminative power of the whole marker set was evaluated using a 10-fold cross-validation on tumor tissues. The discriminative power was quantified as the ROC curve, shown as the bold black curve. The gray area represents the CI of the ROC curve. The AUC was calculated, and the corresponding 95% DeLong CI is shown in the parentheses.

To identify the methylation markers for the two lung cancer subtypes, we collected RRBS data for 60 solid tissues for marker discovery, including 31 tumor samples from patients with lung adenocarcinoma and 29 tumor samples from patients with LUSC. Using our fragment-based marker discovery method (see “Material and Methods”), we identified subtype-specific markers that differ significantly between lung adenocarcinoma and LUSC tumor tissues. We also required the markers to have differential methylation between the solid tumors and the cfDNA of 30 noncancer individuals. The cfDNA of noncancer individuals is derived from blood cells and normal tissue cells. Therefore, the methylation markers were distinct between the two lung cancer subtypes as well as between the tumor and noncancer background. We ranked the markers by their robustness (see “Material and Methods”) and selected the top 2,500 hypermethylation markers and hypomethylation markers for lung adenocarcinoma and LUSC, respectively (ties are included), resulting in 10,941 markers for the lung cancer subtype prediction (Fig. 2a).

### Validation of the subtype-specific methylation markers

To ensure that the methylation markers represent the difference between lung adenocarcinoma and LUSC, we validated the reproducibility of these markers using the RRBS data of an independent tissue cohort and the 450K data from the TCGA project. On the independent RRBS dataset, we compared the normalized subtype-specific read counts between the lung adenocarcinoma and LUSC tumor tissue samples; 68.3% of our markers showed subtype-specific methylation patterns (i.e., a fold change of >1.5, Fig. 2B) consistent with the tumor tissue data for marker discovery. On TCGA data, approximately 32.0% of our markers contained at least one probe in the Infinium HumanMethylation 450K microarray because of the difference in experimental platforms. The methylation level of these markers in TCGA data is illustrated in Fig. 2C. In this large independent cohort, 68.7% of the markers had a statistically significant subtype-specific methylation pattern (Benjamini–Hochberg adjusted *P* value > 0.05) consistent with the tumor tissue data for marker discovery. These results demonstrate that our methylation markers captured the subtype-specific methylation difference that was mostly reproducible in other patient cohorts.

The potential biological functions related to our methylation markers were then investigated. Using RNA-seq data from the TCGA project, we analyzed the transcription level of a gene if its promoter overlapped with the markers. Approximately 35.8% of the subtype-specific markers (*n* = 3,918) overlapped with the promoter region of genes included in TCGA RNA-seq data. These markers corresponded to 2,996 genes. From the RNA-seq data, we observed different transcription levels between lung adenocarcinoma and LUSC (Fig. 2D). There was a significant difference in the transcription levels of 44.3% of the genes (Fig. 2E). This fraction of genes was significantly larger than the random genes (empirical *P* value = 0). These results suggest that our methylation markers likely impact downstream subtype-specific transcription activity.

We assessed the overall discriminative power of the marker set in distinguishing between lung adenocarcinoma and LUSC. With individual methylation markers validated in the independent cohorts, we built a logistic regression classification model using the solid tumor samples from the patients with lung adenocarcinoma and LUSC in the TCGA project. Using 435 lung adenocarcinoma and 359 LUSC solid tumor samples, we used the methylation levels at each marker as features. We used a 10-fold cross-validation approach and divided samples into 10 folds, training the model on nine folds and testing it on the remaining fold. Across 10 folds, our methylation markers achieved an AUC of 0.987 [95% DeLong confidence interval (CI) = (0.979, 0.994), Fig. 2F], indicating a high distinctive power of our marker set.

### Distinguishing lung cancer subtypes using plasma cfDNA

After validating the subtype-specific markers, we next evaluated whether lung cancer subtypes can be identified from cfDNA methylation. Because tumor cells from all tumor sites and clones can release DNA into the bloodstream, cfDNA potentially contains a more comprehensive profile of the heterogeneous tumor than a tumor sample biopsied at a single tumor site (14, 15). Considering the heterogeneity of lung cancer, subtyping lung cancers with cfDNA holds promise in cancer detection and monitoring. We generated *cfMethyl-Seq* data of the plasma cfDNA samples collected from 66 patients with lung adenocarcinoma and 40 patients with LUSC. Using the data from a patient cfDNA sample, we extracted the cfDNA fragment–based methylation patterns at the markers. Fragment-based methylation patterns can separate the cfDNA fragments carrying the subtype-specific methylation from the leukocyte-derived background, enhancing weak tumor signals in the cfDNA with low tumor content (9).

**Figure 3:**
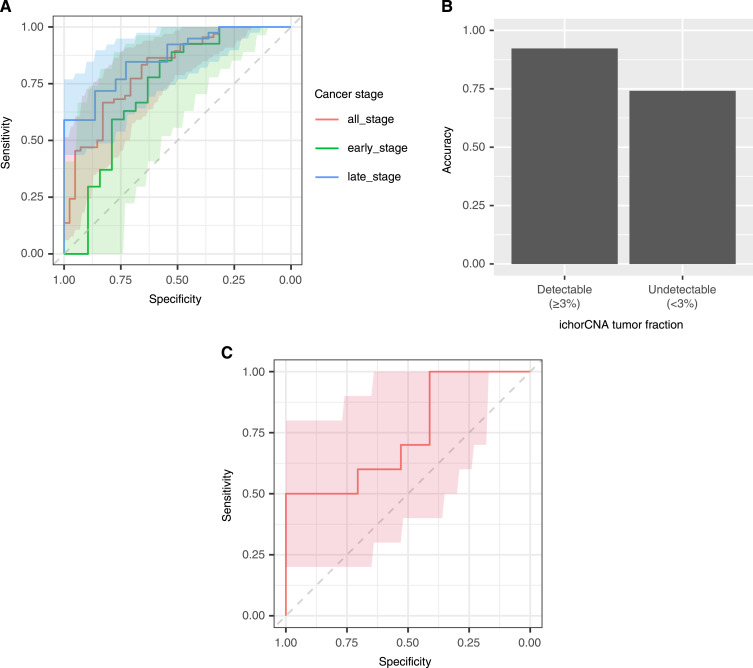
Classification of lung cancer subtypes using plasma cfDNA. **A,** ROC curve of subtype classification on plasma cfDNA. The performance of the subtype classification model was evaluated using LOOCV. The bold curves are the ROC curve. The colored areas represent the CI of the ROC curve. **B,** Accuracy of the subtype classification. Accuracy was evaluated on the plasma samples with detectable and undetectable tumor fractions, as estimated using ichorCNA. **C,** ROC curve of subtype classification on an independent validation set.

Utilizing the extracted fragment-based methylation profiles, we built a logistic regression classification model to distinguish lung adenocarcinoma and LUSC using the cfDNA. The model was first evaluated using LOOCV. Our methylation markers achieved an AUC of 0.808 [95% DeLong CI = (0.722, 0.893), accuracy = 0.764, Fig. 3A] across all stages, an AUC of 0.729 [95% DeLong CI = (0.565, 0.893), accuracy = 0.739] for stages I to II patients (*n* = 46), and an AUC of 0.867 [95% DeLong CI = (0.780, 0.954), accuracy = 0.783] for stages III to IV patients (*n* = 60). We then evaluated the classification accuracy in the plasma samples based on their tumor fractions. We estimated the tumor fraction and identified tumor copy-number alterations from the *cfMethyl-Seq* data using ichorCNA (31). Because the detection limit of ichorCNA is at a tumor fraction of approximately 3% (31), we divided the plasma samples into two groups, with detectable tumor fractions (≥3%) and undetectable tumor fractions (<3%). The accuracy of subtype classification was higher in the plasma samples with a detectable tumor fraction (*n* = 13, accuracy = 92.3%) than in the plasma samples with an undetectable tumor fraction (*n* = 93, accuracy = 74.2%, Fig. 3B). The tumor copy-number alteration analysis on cfDNA methylome revealed that 4 of the 13 patients with detectable tumor fractions showed chromosome 9p21.3 loss (Supplementary Fig. S2), a known indicator associated with profound immune-cold tumors (32). This suggests that tumor aneuploidies inferred from cfDNA methylome may potentially help inform personalized treatment selection for immunotherapy.

We evaluated the performance of this cfDNA-based classification model using an independent *cfMethyl-Seq* dataset derived from plasma cfDNA samples collected from 17 patients with lung adenocarcinoma and 10 patients with LUSC. The model achieved an AUC of 0.747 [95% DeLong CI = (0.543, 0.951), accuracy = 0.704, Fig. 3C] in this independent validation. The model performance for different cancer stages was not evaluated because of the limited sample size. In this independent validation set, three patients had detectable tumor fractions by ichorCNA, one of whom showed chromosome 9p21.3 loss (Supplementary Fig. S3). Despite the small sample size and the limited sequencing depth of these *cfMethyl-Seq* data (median 22.6×), these results demonstrate the potential of noninvasively subtyping lung cancers and detecting tumor molecular characteristics using cfDNA methylome.

## Discussion

Accurate classification of histologic subtypes of lung cancers is essential for diagnosis, treatment, and prognosis. The current practice of tumor biopsy provides standard classification of the histologic subtypes. With the emerging potential of noninvasive cancer detection and typing through cfDNA, we explored the feasibility of utilizing cfDNA for the noninvasive classification of histologic subtypes in lung cancers. We focused on the two most prevalent subtypes of lung cancer, lung adenocarcinoma and LUSC, and developed one of the first cfDNA methylation–based classification models. This method provides a supplemental strategy for lung cancer subtyping.

To build this classification model, we systematically examined the methylation profiles of 31 lung adenocarcinoma and 29 LUSC tumor tissues. We identified differentially methylated markers using a fragment-based marker discovery strategy, which overcomes the impure and heterogeneous signals from the tumor samples. These markers were successfully reproduced in two independent patient cohorts, which indicated the robust methylation difference between the two subtypes. By investigating the RNA-seq data from TCGA, we found that 44.3% of genes associated with the methylation markers had a significant differential transcription level. These results demonstrate that our methylation markers are related to the differential gene expression signatures characteristic of the two subtypes. We further demonstrated the ability of the markers to distinguish between lung adenocarcinoma and LUSC, achieving an AUC of 0.987 in the 10-fold cross-validation using tumor tissue data from TCGA.

Based on these markers, we built a logistic regression model to classify plasma samples from the patients with lung adenocarcinoma and LUSC based on the cfDNA methylation profiles. The plasma samples were profiled with *cfMethyl-Seq* at a limited depth (median 22.6×). With these data, we achieved an AUC of 0.808 using LOOCV and an AUC of 0.747 in the independent validation. This performance is consistent with existing studies of lung cancer subtyping using cfDNA fragmentation patterns (34) and cfDNA methylation at a limited number of genes (35). Apart from them, another epigenetic molecular marker, transcription factor binding inferred from cfDNA fragmentation patterns, has been used in classifying lung cancer subtypes with promising accuracy (36). *cfMethyl-Seq* retains the genome-wide methylation profiles of cancer abnormalities in a cost-effective manner, allowing the classification model to learn and exploit newly identified significant features as the training cohorts expand (9). With a higher sequencing depth and larger training size, we anticipate the classification performance to further improve.

Our tumor copy-number alteration analysis on cfDNA methylome data derived from plasma cfDNA samples identified chromosome 9p21.3 loss in a total of 5 of 16 patients with non–small cell lung cancer with detectable tumor fractions (≥3%; 4 of 13 in the training cohort and 1 of 3 in the validation cohort), including 4 of 12 patients with lung adenocarcinoma (33%) and 1 of 4 patients with LUSC (25%). Among chromosome 9p alterations, 9p21.3 homozygous deletions (9p21.3 loss) are among the most prevalent events of somatic copy-number alterations (SCNA) occurring in ∼13% of all cancers, including ∼20% of human papillomavirus–negative head and neck cancers, ∼15% of lung adenocarcinoma, and ∼25% of LUSC (32, 33, 37). Chromosome 9p21.3 loss eliminates *CDKN2A/B* tumor suppressors and often encompasses codeletions of a cluster of 16 type I *IFN* genes (32, 33, 37). Recent studies demonstrate that 9p21.3 loss is associated with a type I *IFN*–mediated immune-cold tumor phenotype and resistance to immune checkpoint inhibitors (32, 33, 37, 38). Our cfDNA methylome platform therefore has the potential to identify SCNAs noninvasively and facilitate personalized and optimal treatment decisions for each patient. Although the sample size is small, we detected a higher percentage of patients with lung adenocarcinoma harboring chromosome 9p21.3 loss than that identified by TCGA datasets (33, 37), suggesting that genomic data derived from cfDNA secreted by all tumor cells may enhance the probability of detecting SCNAs compared with data generated from tumor biopsies. Future studies with larger patient cohorts will offer validation about the detection of cancer aneuploidies by cfDNA methylome.

In summary, we identified robust differential methylation markers with strong distinguishing power between lung adenocarcinoma and LUSC and developed a classification model to noninvasively distinguish these subtypes using cfDNA methylation. Our cfDNA methylome analysis has the potential for noninvasive early detection as well as cancer monitoring. It enables longitudinal cancer monitoring by tracking lung adenocarcinoma and LUSC progression during treatment, potentially indicating either treatment response or resistance. The tumor copy-number alterations identified from the cfDNA methylome data can offer additional insights for treatment selection. For early detection, the platform seamlessly integrates into existing workflows (9), providing preliminary histologic subtype information to complement current cancer detection and tissue origin assessment. For example, the use of cfDNA methylome analysis could be implemented in the context of lung cancer screening with low-dose CT scanning to aid in the diagnosis of indeterminant pulmonary nodules. Our primary focus on the two most prevalent subtypes of non–small cell lung cancer serves as a promising example. Future studies may extend this method to encompass other subtypes when adequate data are available for marker discovery and model training. This cost-effective genome-wide methylation profiling utilizing cfDNA methylome analysis holds promise for the enhancement of early cancer detection and cancer monitoring.

## Supplementary Material

Supplementary Figure 1 Fragment-based marker discovery between LUAD and LUSC

Supplementary Figure 2 Tumor copy number variations identified from 106 cfDNA samples for model training and cross-validation

Supplementary Figure 3 Tumor copy number variations identified from 27 cfDNA samples for independent validation

Supplementary Table 1 Clinical information of the tissue and blood samples
