# Supplementary material for: Noninvasive Lung Cancer Subtype Classification Using Tumor-Derived Signatures and cfDNA Methylome
Source: Cancer Res Commun. 2024 Jul 16;4(7):1738–47. doi: 10.1158/2767-9764.CRC-23-0564 (PMC11249519; doi:10.1158/2767-9764.CRC-23-0564)

Supplementary Figure 1. Fragment-based marker discovery between LUAD and LUSC. (a) The α-value distribution of a genomic region. Each line represents a DNA fragment and each dot represents a CpG site, where solid blue dots represent methylated CpG sites, and hollow blue dots represent unmethylated CpG sites. The α-value of a DNA fragment was calculated as the fraction of methylated CpG sites in all CpG sites in this DNA fragment, horizontal direction). The α-value distribution of a genomic region is the distribution of the α-value of all DNA fragments mapped to this region. **(b)** An example of fragment-based marker discovery. Using the α-value distributions of the tumor tissues from the LUAD and LUSC patients, we can identify a threshold (e.g. $\alpha_{\text{hypo}} = 0.5$) to define hypomethylated reads that occur mostly in the tumor tissues of the LUAD patients but not in the LUSC patients. To select markers, we count the number of tumor tissues from the LUAD patients that have hypomethylated reads as $n_{\text{hypo}}^{\text{LUAD}}$ and the number of tumor tissues from the LUSC patients that have hypomethylated reads as $n_{\text{hypo}}^{\text{LUSC}}$. The $n_{\text{hypo}}^{\text{LUSC}}$ and $n_{\text{hypo}}^{\text{LUAD}}$ are then used to filter and rank the genomic regions for marker selection.

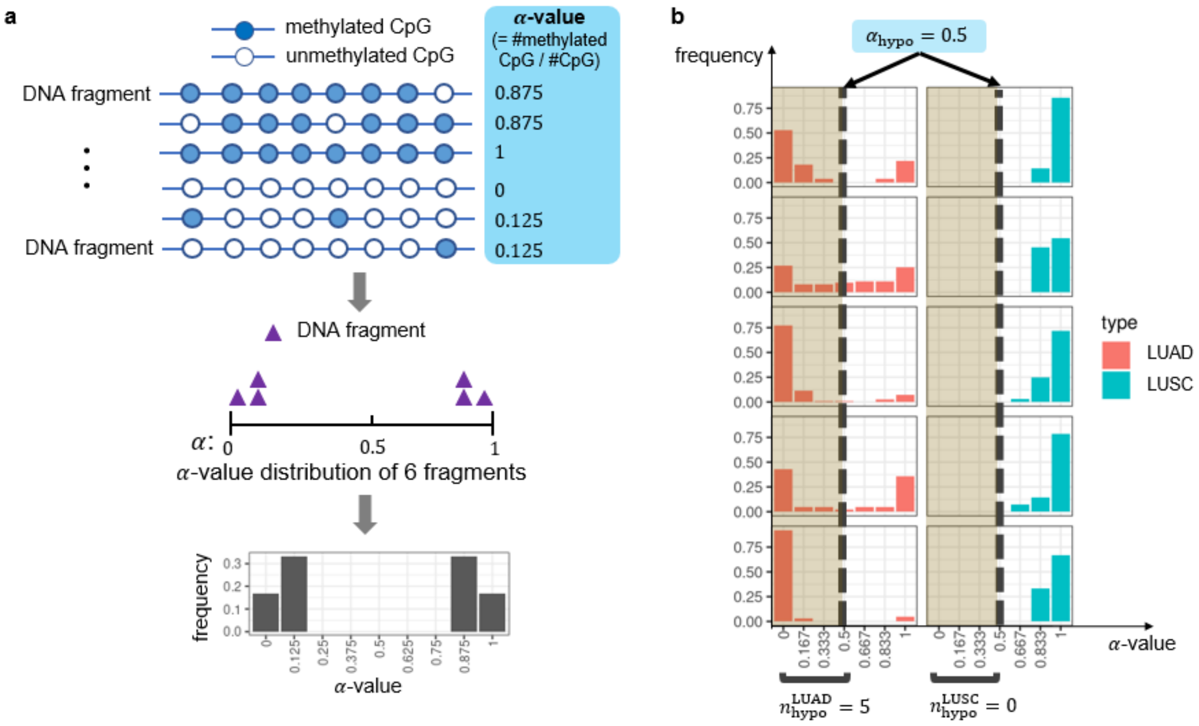

Supplement: Supplementary Figure 1 — Fragment-based marker discovery between LUAD and LUSC [file crc-23-0564_supplementary_figure_1_suppsf1.pdf]
