# Supplementary material for: Noninvasive Lung Cancer Subtype Classification Using Tumor-Derived Signatures and cfDNA Methylome
Source: Cancer Res Commun. 2024 Jul 16;4(7):1738–47. doi: 10.1158/2767-9764.CRC-23-0564 (PMC11249519; doi:10.1158/2767-9764.CRC-23-0564)

Supplementary Figure 2. Tumor copy number variations identified from 106 cfDNA samples for model training and cross-validation. We employed ichorCNA to detect tumor copy number variations from the methylome data. The genome was segmented into 1 million base-pair bins and a reference panel was created from 30 noncancer plasma samples. Every dot in the plot reflects the estimated copy number in the cfDNA in the corresponding 1-million base-pair bin.

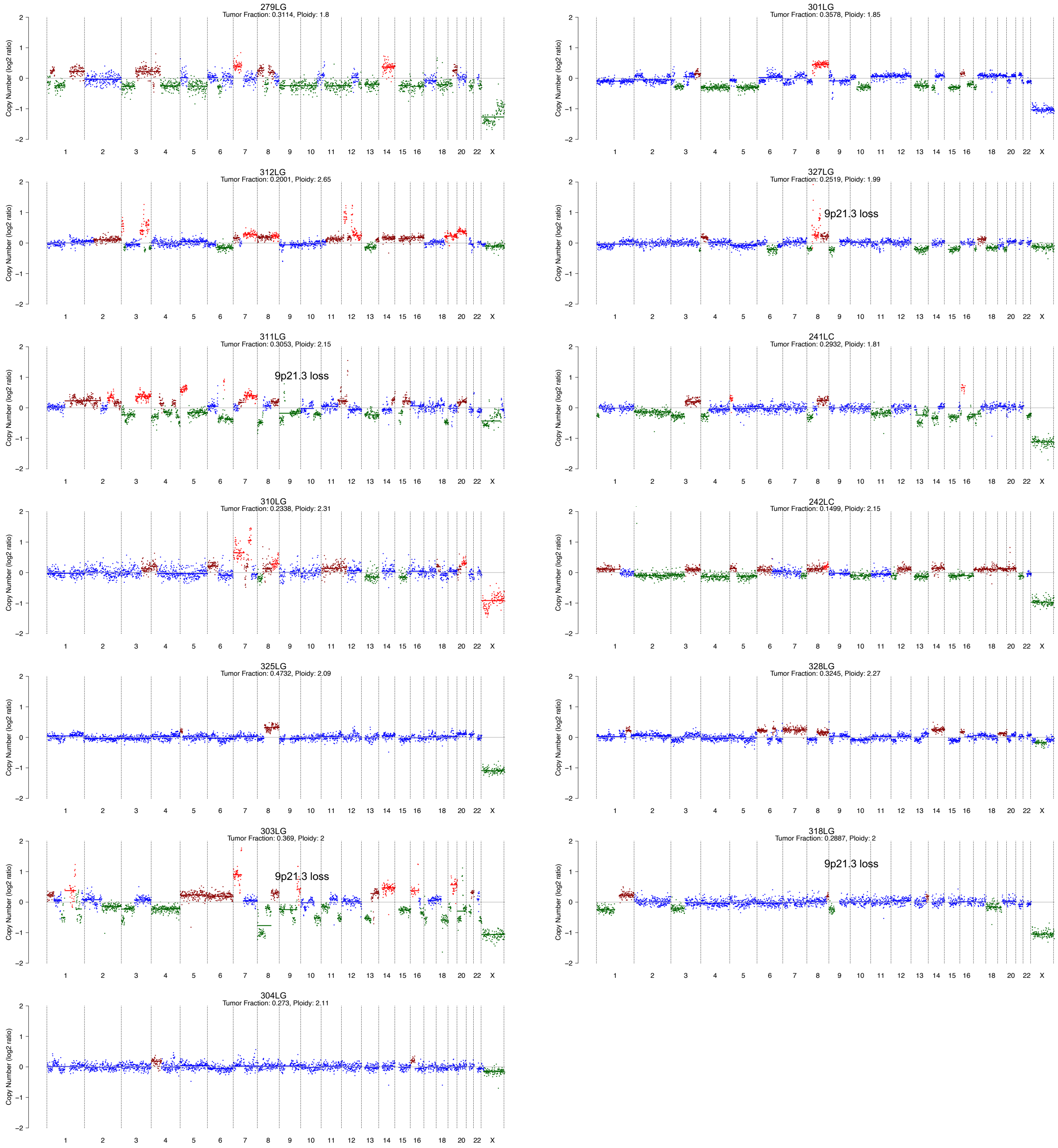

Supplement: Supplementary Figure 2 — Tumor copy number variations identified from 106 cfDNA samples for model training and cross-validation [file crc-23-0564_supplementary_figure_2_suppsf2.pdf]
