# Supplementary material for: Noninvasive Lung Cancer Subtype Classification Using Tumor-Derived Signatures and cfDNA Methylome
Source: Cancer Res Commun. 2024 Jul 16;4(7):1738–47. doi: 10.1158/2767-9764.CRC-23-0564 (PMC11249519; doi:10.1158/2767-9764.CRC-23-0564)

Supplementary Figure 3. Tumor copy number variations identified from 27 cfDNA samples for independent validation. We employed ichorCNA to detect tumor copy number variations from the methylome data. The genome was segmented into 1 million base-pair bins and a reference panel was created from 30 noncancer plasma samples. Every dot in the plot reflects the estimated copy number in the cfDNA in the corresponding 1-million base-pair bin.

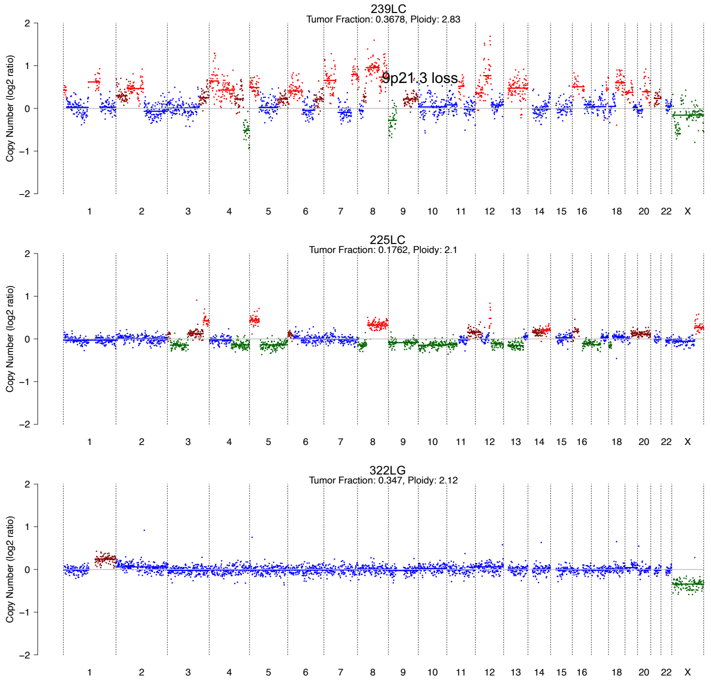

Supplement: Supplementary Figure 3 — Tumor copy number variations identified from 27 cfDNA samples for independent validation [file crc-23-0564_supplementary_figure_3_suppsf3.pdf]
