# Supplementary material for: Noninvasive Lung Cancer Subtype Classification Using Tumor-Derived Signatures and cfDNA Methylome
Source: Cancer Res Commun. 2024 Jul 16;4(7):1738–47. doi: 10.1158/2767-9764.CRC-23-0564 (PMC11249519; doi:10.1158/2767-9764.CRC-23-0564)
Supplement: Supplementary Table 1 — Clinical information of the tissue and blood samples [file crc-23-0564_supplementary_table_1_suppst1.pdf]

Supplementary Table 1. Clinical information of the tissue and blood samples. The sample ID is consistent with the published data in Stackpole ML et al.

sample_id	cancer_subtype	stage	gender	usage	biospecimen
504LG	LUADå	IA	F	independent validation	plasma cfDNA
508LG	LUAD	IB	M	independent validation	plasma cfDNA
509LG	LUAD	IB	M	independent validation	plasma cfDNA
505LG	LUAD	IA	M	independent validation	plasma cfDNA
259LG	LUAD	IA	M	independent validation	plasma cfDNA
510LG	LUAD	IIIA	M	independent validation	plasma cfDNA
506LG	LUAD	IA	F	independent validation	plasma cfDNA
226LC	LUSC	IIB	M	independent validation	plasma cfDNA
239LC	LUSC	IIIA	F	independent validation	plasma cfDNA
205LC	LUSC	IA	M	independent validation	plasma cfDNA
225LC	LUSC	IIB	M	independent validation	plasma cfDNA
500LC	LUSC	IIIA	M	independent validation	plasma cfDNA
216LC	LUSC	IIA	M	independent validation	plasma cfDNA
220LC	LUSC	IIB	M	independent validation	plasma cfDNA
511LG	LUAD	IIIA	F	independent validation	plasma cfDNA
261LG	LUAD	IA	M	independent validation	plasma cfDNA
275LG	LUAD	IB	F	independent validation	plasma cfDNA
248LC	LUSC	IV	M	independent validation	plasma cfDNA
322LG	LUAD	IV	F	independent validation	plasma cfDNA
502LC	LUSC	IV	M	independent validation	plasma cfDNA
323LG	LUAD	IV	M	independent validation	plasma cfDNA
503LG	LUAD	unknown	unknown	independent validation	plasma cfDNA
501LC	LUSC	IV	M	independent validation	plasma cfDNA
513LG	LUAD	IIIA	M	independent validation	plasma cfDNA
507LG	LUAD	IB	M	independent validation	plasma cfDNA
514LG	LUAD	IIIB	M	independent validation	plasma cfDNA
512LG	LUAD	IIIA	F	independent validation	plasma cfDNA
2N	normal	NA	M	marker discovery	plasma cfDNA
8N	normal	NA	M	marker discovery	plasma cfDNA
13N	normal	NA	M	marker discovery	plasma cfDNA
22N	normal	NA	M	marker discovery	plasma cfDNA
24N	normal	NA	M	marker discovery	plasma cfDNA
40N	normal	NA	M	marker discovery	plasma cfDNA
46N	normal	NA	M	marker discovery	plasma cfDNA
47N	normal	NA	F	marker discovery	plasma cfDNA
58N	normal	NA	M	marker discovery	plasma cfDNA
76N	normal	NA	M	marker discovery	plasma cfDNA
80N	normal	NA	M	marker discovery	plasma cfDNA
86N	normal	NA	M	marker discovery	plasma cfDNA

88N	normal	NA	F	marker discovery	plasma cfDNA
92N	normal	NA	F	marker discovery	plasma cfDNA
93N	normal	NA	M	marker discovery	plasma cfDNA
99N	normal	NA	F	marker discovery	plasma cfDNA
101N	normal	NA	M	marker discovery	plasma cfDNA
110N	normal	NA	M	marker discovery	plasma cfDNA
112N	normal	NA	F	marker discovery	plasma cfDNA
113N	normal	NA	F	marker discovery	plasma cfDNA
131N	normal	NA	M	marker discovery	plasma cfDNA
139N	normal	NA	F	marker discovery	plasma cfDNA
141N	normal	NA	F	marker discovery	plasma cfDNA
159N	normal	NA	F	marker discovery	plasma cfDNA
173N	normal	NA	F	marker discovery	plasma cfDNA
174N	normal	NA	F	marker discovery	plasma cfDNA
176N	normal	NA	F	marker discovery	plasma cfDNA
186N	normal	NA	M	marker discovery	plasma cfDNA
187N	normal	NA	F	marker discovery	plasma cfDNA
195N	normal	NA	M	marker discovery	plasma cfDNA
298LG	LUAD	IIIB	M	model training	plasma cfDNA
315LG	LUAD	IV	M	model training	plasma cfDNA
277LG	LUAD	IB	F	model training	plasma cfDNA
260LG	LUAD	IA	F	model training	plasma cfDNA
262LG	LUAD	IA	F	model training	plasma cfDNA
256LG	LUAD	IA	M	model training	plasma cfDNA
291LG	LUAD	IIIA	M	model training	plasma cfDNA
276LG	LUAD	IB	M	model training	plasma cfDNA
283LG	LUAD	IIA	F	model training	plasma cfDNA
306LG	LUAD	IV	F	model training	plasma cfDNA
279LG	LUAD	IB	M	model training	plasma cfDNA
281LG	LUAD	IB	F	model training	plasma cfDNA
293LG	LUAD	IIIA	F	model training	plasma cfDNA
215LC	LUSC	IIA	M	model training	plasma cfDNA
207LC	LUSC	IA	M	model training	plasma cfDNA
244LC	LUSC	IV	M	model training	plasma cfDNA
214LC	LUSC	IIA	M	model training	plasma cfDNA
217LC	LUSC	IIA	M	model training	plasma cfDNA
230LC	LUSC	IIIA	M	model training	plasma cfDNA
222LC	LUSC	IIB	M	model training	plasma cfDNA
240LC	LUSC	IIIB	M	model training	plasma cfDNA
223LC	LUSC	IIB	M	model training	plasma cfDNA
250LC	LUSC	IV	F	model training	plasma cfDNA
236LC	LUSC	IIIA	M	model training	plasma cfDNA
247LC	LUSC	IV	F	model training	plasma cfDNA

224LC	LUSC	IIB	M	model training	plasma cfDNA
228LC	LUSC	IIB	M	model training	plasma cfDNA
211LC	LUSC	IIA	M	model training	plasma cfDNA
232LC	LUSC	IIIA	M	model training	plasma cfDNA
212LC	LUSC	IIA	M	model training	plasma cfDNA
287LG	LUAD	IIIA	F	model training	plasma cfDNA
263LG	LUAD	IA	M	model training	plasma cfDNA
312LG	LUAD	IV	F	model training	plasma cfDNA
330LG	LUAD	IV	M	model training	plasma cfDNA
308LG	LUAD	IV	M	model training	plasma cfDNA
329LG	LUAD	IV	M	model training	plasma cfDNA
311LG	LUAD	IV	F	model training	plasma cfDNA
309LG	LUAD	IV	M	model training	plasma cfDNA
302LG	LUAD	IV	F	model training	plasma cfDNA
305LG	LUAD	IV	M	model training	plasma cfDNA
324LG	LUAD	IV	F	model training	plasma cfDNA
252LC	LUSC	IV	M	model training	plasma cfDNA
325LG	LUAD	IV	M	model training	plasma cfDNA
303LG	LUAD	IV	M	model training	plasma cfDNA
246LC	LUSC	IV	M	model training	plasma cfDNA
326LG	LUAD	IV	M	model training	plasma cfDNA
307LG	LUAD	IV	M	model training	plasma cfDNA
316LG	LUAD	IV	M	model training	plasma cfDNA
313LG	LUAD	IV	M	model training	plasma cfDNA
304LG	LUAD	IV	F	model training	plasma cfDNA
320LG	LUAD	IV	F	model training	plasma cfDNA
301LG	LUAD	IV	M	model training	plasma cfDNA
327LG	LUAD	IV	F	model training	plasma cfDNA
321LG	LUAD	IV	M	model training	plasma cfDNA
227LC	LUSC	IIB	M	model training	plasma cfDNA
213LC	LUSC	IIA	M	model training	plasma cfDNA
206LC	LUSC	IA	M	model training	plasma cfDNA
234LC	LUSC	IIIA	M	model training	plasma cfDNA
245LC	LUSC	IV	M	model training	plasma cfDNA
237LC	LUSC	IIIA	M	model training	plasma cfDNA
221LC	LUSC	IIB	M	model training	plasma cfDNA
210LC	LUSC	IIA	M	model training	plasma cfDNA
238LC	LUSC	IIIA	M	model training	plasma cfDNA
219LC	LUSC	IIB	M	model training	plasma cfDNA
235LC	LUSC	IIIA	F	model training	plasma cfDNA
231LC	LUSC	IIIA	M	model training	plasma cfDNA
243LC	LUSC	IIIB	M	model training	plasma cfDNA
241LC	LUSC	IIIB	M	model training	plasma cfDNA

242LC	LUSC	IIIB	M	model training	plasma cfDNA
253LC	LUSC	IV	F	model training	plasma cfDNA
249LC	LUSC	IV	M	model training	plasma cfDNA
208LC	LUSC	IA	M	model training	plasma cfDNA
274LG	LUAD	IB	F	model training	plasma cfDNA
280LG	LUAD	IB	F	model training	plasma cfDNA
288LG	LUAD	IIIA	M	model training	plasma cfDNA
209LC	LUSC	IB	M	model training	plasma cfDNA
314LG	LUAD	IV	F	model training	plasma cfDNA
286LG	LUAD	IIIA	F	model training	plasma cfDNA
295LG	LUAD	IIIA	M	model training	plasma cfDNA
328LG	LUAD	IV	F	model training	plasma cfDNA
310LG	LUAD	IV	M	model training	plasma cfDNA
317LG	LUAD	IV	F	model training	plasma cfDNA
318LG	LUAD	IV	M	model training	plasma cfDNA
319LG	LUAD	IV	M	model training	plasma cfDNA
268LG	LUAD	IA2	F	model training	plasma cfDNA
266LG	LUAD	IA1	F	model training	plasma cfDNA
282LG	LUAD	IB	F	model training	plasma cfDNA
272LG	LUAD	IA2	F	model training	plasma cfDNA
265LG	LUAD	IA1	F	model training	plasma cfDNA
270LG	LUAD	IA2	F	model training	plasma cfDNA
269LG	LUAD	IA2	F	model training	plasma cfDNA
284LG	LUAD	IIA	M	model training	plasma cfDNA
300LG	LUAD	IIIB	M	model training	plasma cfDNA
233LC	LUSC	IIIA	M	model training	plasma cfDNA
290LG	LUAD	IIIA	M	model training	plasma cfDNA
296LG	LUAD	IIIA	M	model training	plasma cfDNA
285LG	LUAD	IIB	F	model training	plasma cfDNA
258LG	LUAD	IA	M	model training	plasma cfDNA
289LG	LUAD	IIIA	M	model training	plasma cfDNA
257LG	LUAD	IA3	F	model training	plasma cfDNA
254LG	LUAD	IA1	M	model training	plasma cfDNA
267LG	LUAD	IA2	M	model training	plasma cfDNA
271LG	LUAD	IA2	M	model training	plasma cfDNA
218LC	LUSC	IIA	M	model training	plasma cfDNA
273LG	LUAD	IA3	M	model training	plasma cfDNA
255LG	LUAD	IIB	F	model training	plasma cfDNA
lung_T-138-LG	LUAD	IIIA	F	marker discovery	tumor gDNA
lung_T-1-LG	LUAD	IA	F	marker discovery	tumor gDNA
lung_T-2-LG	LUAD	IV	F	marker discovery	tumor gDNA
lung_T-10-LG	LUAD	IIB	F	marker discovery	tumor gDNA
lung_T-13-LG	LUAD	IIIA	M	marker discovery	tumor gDNA

lung_T-15-LG	LUAD	IIIA	M	marker discovery	tumor gDNA
lung_T-16-LC	LUSC	IIB	F	marker discovery	tumor gDNA
lung_T-17-LG	LUAD	IA	M	marker discovery	tumor gDNA
lung_T-20-LG	LUAD	IB	F	marker discovery	tumor gDNA
lung_T-21-LC	LUSC	IIIA	M	marker discovery	tumor gDNA
lung_T-22-LG	LUAD	IIA	M	marker discovery	tumor gDNA
lung_T-23-LG	LUAD	IV	F	marker discovery	tumor gDNA
lung_T-24-LG	LUAD	IB	F	marker discovery	tumor gDNA
lung_T-31-LG	LUAD	IIIA	M	marker discovery	tumor gDNA
lung_T-32-LG	LUAD	IIB	F	marker discovery	tumor gDNA
lung_T-33-LG	LUAD	IB	F	marker discovery	tumor gDNA
lung_T-34-LG	LUAD	IIB	F	marker discovery	tumor gDNA
lung_T-139-LC	LUSC	IIIA	M	marker discovery	tumor gDNA
lung_T-35-LC	LUSC	IA	M	marker discovery	tumor gDNA
lung_T-37-LC	LUSC	IIIB	F	marker discovery	tumor gDNA
lung_T-38-LC	LUSC	IA	F	marker discovery	tumor gDNA
lung_T-39-LC	LUSC	IB	M	marker discovery	tumor gDNA
lung_T-40-LC	LUSC	IB	F	marker discovery	tumor gDNA
lung_T-45-LC	LUSC	IB	M	marker discovery	tumor gDNA
lung_T-46-LC	LUSC	unknown	M	marker discovery	tumor gDNA
lung_T-47-LC	LUSC	IB	M	marker discovery	tumor gDNA
lung_T-48-LC	LUSC	IB	M	marker discovery	tumor gDNA
lung_T-49-LC	LUSC	IB	F	marker discovery	tumor gDNA
lung_T-50-LC	LUSC	IB	M	marker discovery	tumor gDNA
lung_T-51-LC	LUSC	IA	M	marker discovery	tumor gDNA
lung_T-52-LC	LUSC	IB	M	marker discovery	tumor gDNA
lung_T-243-LG	LUAD	IIIA	F	marker discovery	tumor gDNA
lung_T-77-LG	LUAD	IB	F	marker discovery	tumor gDNA
lung_T-78-LG	LUAD	IIIA	M	marker discovery	tumor gDNA
lung_T-79-LG	LUAD	IB	M	marker discovery	tumor gDNA
lung_T-80-LG	LUAD	IB	M	marker discovery	tumor gDNA
lung_T-83-LC	LUSC	IIIB	M	marker discovery	tumor gDNA
lung_T-84-LC	LUSC	III	F	marker discovery	tumor gDNA
lung_T-85-LC	LUSC	I	M	marker discovery	tumor gDNA
lung_T-86-LC	LUSC	IIB	M	marker discovery	tumor gDNA
lung_T-87-LC	LUSC	IB	F	marker discovery	tumor gDNA
lung_T-88-LC	LUSC	IB	M	marker discovery	tumor gDNA
lung_T-89-LC	LUSC	IB	F	marker discovery	tumor gDNA
lung_T-99-LG	LUAD	IIA	M	marker discovery	tumor gDNA
lung_T-100-LG	LUAD	IA	F	marker discovery	tumor gDNA
lung_T-101-LG	LUAD	IIB	M	marker discovery	tumor gDNA
lung_T-244-LG	LUAD	IA	F	marker discovery	tumor gDNA
lung_T-245-LG	LUAD	IA	F	marker discovery	tumor gDNA

lung_T-246-LC	LUSC	IIIA	M	marker discovery	tumor gDNA
lung_T-247-LC	LUSC	IA	F	marker discovery	tumor gDNA
lung_T-248-LC	LUSC	IIB	M	marker discovery	tumor gDNA
lung_T-252-LG	LUAD	IA1	M	marker discovery	tumor gDNA
lung_T-253-LG	LUAD	IA1	F	marker discovery	tumor gDNA
lung_T-254-LC	LUSC	IB	M	marker discovery	tumor gDNA
lung_T-255-LC	LUSC	IA	M	marker discovery	tumor gDNA
lung_T-256-LG	LUAD	IA	F	marker discovery	tumor gDNA
lung_T-257-LG	LUAD	IA	F	marker discovery	tumor gDNA
lung_T-249-LG	LUAD	IA2	M	marker discovery	tumor gDNA
lung_T-250-LG	LUAD	IA1	F	marker discovery	tumor gDNA
lung_T-251-LC	LUSC	IA1	M	marker discovery	tumor gDNA
lung_T-200-LG	LUAD	IB	M	marker validation	tumor gDNA
lung_T-201-LG	LUAD	IB	F	marker validation	tumor gDNA
lung_T-202-LG	LUAD	IA	F	marker validation	tumor gDNA
lung_T-203-LG	LUAD	IIIA	M	marker validation	tumor gDNA
lung_T-204-LG	LUAD	IIB	M	marker validation	tumor gDNA
lung_T-205-LG	LUAD	IIIA	M	marker validation	tumor gDNA
lung_T-206-LG	LUAD	IIB	M	marker validation	tumor gDNA
lung_T-207-LG	LUAD	IIA	M	marker validation	tumor gDNA
lung_T-208-LG	LUAD	IB	M	marker validation	tumor gDNA
lung_T-227-LG	LUAD	IB	F	marker validation	tumor gDNA
lung_T-209-LC	LUSC	IB	M	marker validation	tumor gDNA
lung_T-210-LC	LUSC	IB	F	marker validation	tumor gDNA
lung_T-211-LC	LUSC	IA	M	marker validation	tumor gDNA
lung_T-212-LC	LUSC	IIB	F	marker validation	tumor gDNA
lung_T-213-LC	LUSC	IB	M	marker validation	tumor gDNA
lung_T-214-LC	LUSC	IB	M	marker validation	tumor gDNA
lung_T-226-LG	LUAD	IIIA	F	marker validation	tumor gDNA
lung_T-228-LG	LUAD	IB	F	marker validation	tumor gDNA
lung_T-220-LG	LUAD	III	F	marker validation	tumor gDNA
lung_T-221-LG	LUAD	IIB	F	marker validation	tumor gDNA
lung_T-222-LG	LUAD	IIB	F	marker validation	tumor gDNA
lung_T-223-LG	LUAD	IIIA	F	marker validation	tumor gDNA
lung_T-224-LG	LUAD	IA	F	marker validation	tumor gDNA
lung_T-225-LG	LUAD	IA	F	marker validation	tumor gDNA
lung_T-215-LC	LUSC	I	M	marker validation	tumor gDNA
lung_T-216-LC	LUSC	IIB	M	marker validation	tumor gDNA
lung_T-217-LC	LUSC	IIB	M	marker validation	tumor gDNA
lung_T-218-LG	LUAD	unknown	M	marker validation	tumor gDNA
lung_T-219-LG	LUAD	IIIA	F	marker validation	tumor gDNA
lung_T-229-LG	LUAD	IIA	F	marker validation	tumor gDNA
lung_T-230-LG	LUAD	IIB	F	marker validation	tumor gDNA

lung_T-231-LG	LUAD	IIA	M	marker validation	tumor gDNA
lung_T-232-LG	LUAD	IA	M	marker validation	tumor gDNA
lung_T-233-LC	LUSC	IB	M	marker validation	tumor gDNA
lung_T-234-LC	LUSC	IB	M	marker validation	tumor gDNA
lung_T-235-LC	LUSC	IIB	F	marker validation	tumor gDNA
lung_T-236-LG	LUAD	IA1	F	marker validation	tumor gDNA
lung_T-237-LC	LUSC	IA1	M	marker validation	tumor gDNA
lung_T-238-LG	LUAD	IA1	F	marker validation	tumor gDNA
lung_T-239-LG	LUAD	IA	M	marker validation	tumor gDNA
lung_T-240-LG	LUAD	IA	M	marker validation	tumor gDNA
lung_T-241-LG	LUAD	IA	F	marker validation	tumor gDNA
lung_T-242-LG	LUAD	IA1	M	marker validation	tumor gDNA
